# Anti-Obesity Effects of Soybean Embryo Extract and Enzymatically-Modified Isoquercitrin

**DOI:** 10.3390/biom10101394

**Published:** 2020-09-30

**Authors:** Minsu Kim, Seowoo Im, Yoon keun Cho, Cheoljun Choi, Yeonho Son, Doyoung Kwon, Young-Suk Jung, Yun-Hee Lee

**Affiliations:** 1 College of Pharmacy and Research Institute of Pharmaceutical Sciences, Seoul National University, Seoul 08826, Korea; kims1431@snu.ac.kr (M.K.); isw0619@snu.ac.kr (S.I.); ykc224@naver.com (Y.k.C.); hgg121@snu.ac.kr (C.C.); syh8116@naver.com (Y.S.); 2 College of Pharmacy, Pusan National University, Busan 46241, Korea; doyoung.kwon@pusan.ac.kr (D.K.); youngjung@pusan.ac.kr (Y.-S.J.)

**Keywords:** soy embryo extract, enzymatically-modified isoquercitrin, adipose tissue, anti-obesity, mitochondria, PKA signaling, ATGL

## Abstract

Soy isoflavones are bioactive phytoestrogens with known health benefits. Soybean embryo extract (SEE) has been consumed as a source of isoflavones, mainly daidzein, glycitein, and genistein. While previous studies have reported the anti-obesity effects of SEE, this study investigates their molecular mechanisms and the synergistic effects of co-treatment with SEE and enzymatically modified isoquercitrin (EMIQ). SEE upregulated genes involved in lipolysis and brown adipocyte markers and increased mitochondrial content in differentiated C3H10T1/2 adipocytes in vitro. Next, we use a high-fat diet-induced obesity mouse model to determine the anti-obesity effect of SEE. Two weeks of single or combined treatment with SEE and EMIQ significantly reduced body weight gain and improved glucose tolerance. Mechanistically, SEE treatment increased mitochondrial content and upregulated genes involved in lipolysis in adipose tissue through the cAMP/PKA-dependent signaling pathway. These effects required a cytosolic lipase adipose triglyceride lipase (ATGL) expression, confirmed by an adipocyte-specific ATGL knockout mouse study. Collectively, this study demonstrates that SEE exerts anti-obesity effects through the activation of adipose tissue metabolism and exhibits a synergistic effect of co-treatment with EMIQ. These results improve our understanding of the mechanisms underlying the anti-obesity effects of SEE related to adipose tissue metabolism.

## 1. Introduction

Obesity is defined as an abnormal accumulation of adipose tissue and is associated with complex clinical comorbidities, such as diabetes mellitus and cardiovascular disease [1,2]. Adipose tissue is a central organ that regulates energy homeostasis [3,4]. In general, adipose tissue can be classified into two major types: brown and white adipose tissue [4]. Brown adipose tissue (BAT) contains multi-locular lipid droplets with high mitochondrial content and uniquely expresses uncoupling protein 1 (UCP1) [3]. BAT dissipates energy into heat by the regulation of UCP1-dependent non-shivering thermogenesis [4]. In contrast, white adipose tissue (WAT) stores energy as triglycerides (TG) and mobilizes free fatty acids when energy is needed for systemic use [5]. Liberation of free fatty acids requires lipolysis (hydrolysis of TG), which is mainly regulated by cAMP-dependent protein kinase A (PKA) activation of cytosolic lipase, including adipose triglyceride lipase (ATGL) and hormone-sensitive lipase (HSL) [5]. Researchers have demonstrated that certain pharmacological or physiological conditions, such as β3-adrenergic receptor (β3-AR) agonist treatment and exposure to cold temperature remodel WAT into BAT-like phenotypes [6]. This plasticity of WAT provides a potential therapeutic target to overcome overweight and obesity by increasing energy expenditure [7].

Soy embryo extract (SEE), used as a source of isoflavones in this study, includes a phytoestrogen mixture including daidzein, glycitein, and genistein [8]. Phytoestrogens are plant-derived compounds similar to mammalian estrogen and have the potential to treat obesity and obesity-related metabolic diseases [9]. In relation to adipose tissue biology, soy isoflavones have been involved in adipogenesis [10] and lipid metabolism [9]. For example, genistein is known to have agonist activity of peroxisome proliferator-activated receptor γ (PPARγ), the master transcription factor that regulates adipogenesis [11,12].

Enzymatically modified isoquercitrin (EMIQ) is a mixture of quercetin glycosides with one to seven sugars bound to quercetin [13,14], and the glycoside form of quercetin was designed to increase the bioavailability of quercetin [11]. The EMIQ is made by the enzymatic treatment of rutin [15], which is attached in the order of glucose-rhamnose at position 3 [13]. According to previous reports, EMIQ activates lipid metabolism by regulating the 5′ adenosine monophosphate-activated protein kinase (AMPK) pathway, which facilitates oxidative mitochondrial metabolism in adipose tissue [16].

In this study, we investigate the potential anti-obesity effects of soy embryo extract (SEE), focusing on the cAMP/PKA-induced lipolysis and browning, and the synergistic effects of SEE and EMIQ co-treatment.

## 2. Materials and Methods

### 2.1. Soy Embryo Extract and Enzymatically-Modified Isoquercitrin

Partially purified isoflavones extracted from soy germ (soy embryo extract: SEE) was provided by AmorePacific Corp. (Seoul, Korea) and used as a source of soy isoflavones in the experiments. Soy isoflavone content of SEE was approximately 10% ± 1% on a non-glycoside basis. The composition of isoflavones is 50% daidzin (daidzein), 40% glycitin (glycitein), and 10% genistin (genistein) [8].

Enzymatically-modified isoquercitrin (EMIQ) obtained by enzymatic rutin hydrolysis [15] was provided by AmorePacific Corp. EMIQ contains 96% enzymatic rutin, of which 73% (70% by dry weight) is α-glucosyl isoquercitrin (α-glucosyl isoquercetin) [14].

### 2.2. Cell Cutlure

C3H10T1/2 (American Type Culture Collection (ATCC)) cells were cultured in Dulbecco’s Modified Eagle’s Medium (DMEM, Sigma, St. Louis, MO, USA) containing 10% fetal bovine serum (FBS, Gibco Thermo Fisher Scientific, Waltham, MA, USA) and 1% penicillin/streptomycin (Thermo Fisher, Waltham, MA, USA) under standard conditions (37 °C, 5% CO2). Cell culture and adipogenic differentiation were performed as previously described [17]. As adipocytes were fully differentiated, exposed media were replaced with DMEM containing 10% FBS and incubated overnight, and SEE (80 µg/mL, 160 µg/mL) were treated for 24 h. 4,4-Difluoro-1,3,5,7,8-Pentamethyl-4-Bora-3a,4a-Diaza-s-Indacene (BODIPY 493/503, Invitrogen Thermo Fisher Scientific, Waltham, MA, USA) staining was performed for neutral lipid staining. TG colorimetric assay kit (Cayman Chemical, Ann Arbor, MI, USA) was used to measure intracellular TG contents. Glycerol levels in conditioned media were measured using glycerol reagent (Sigma, St. Louis, MO, USA) according to the manufacturer’s instructions. Red-fluorescent mitochondrion-selective probes MitoTracker Red CMXRos (Thermo Fisher, Waltham, MA, USA) were used to label mitochondrion in live cells.

### 2.3. Animals

All the protocols of animal studies were reviewed and approved by the Institutional Animal Care and Use Committees of Seoul National University (SNU-200408-1, SNU-191025-2). All animal experiments were conducted according to the guidelines for humane care and use of laboratory animals (Ministry of Food and Drug Safety). In vivo experiments were accomplished at Seoul National University, where mice were housed at 22 ± 1 °C, 12-h light/12-h dark cycle condition with free access to food and water. C57BL/6 mice (six weeks old, male) were purchased from Joongah Bio (Suwon, Gyeonggi-do, Korea). For adipocyte-specific ATGL knockout (KO), Adipoq-CreER (Jackson Laboratory stock# 024671,) mice were crossed with Pnpla2-floxed mice (Jackson Laboratory stock# 024278) [18]. Mice were treated with tamoxifen (Sigma, St. Louis, MO, USA, 75 mg/kg/day) by oral gavage for 5 days to induce Cre recombination-based ATGL KO. Vehicle treated control groups were included. Male mice were used in all experiments.

For the diet-induced obesity model, mice at 8 weeks of age were fed with a 60% fat diet (Research Diets # D12492, protein: 20% kcal, fat: 60% kcal, carbohydrate: 20% kcal, energy density: 5.21 kcal/g) for eight weeks. Mice were then divided into groups and were orally administered either SEE (400 mg/kg body weight), EMIQ (100 mg/kg body weight), or a combination dissolved in distilled water once a day for two weeks. Mice were continuously fed with a high-fat diet during the period of administration. Control groups were treated with distilled water as vehicle. As a control chow diet, a standardized rodent pellet diet (Cat. No. 38057; Purina feed, Sung-Nam, Gyeonggi-do, Korea) was used. Details of diet components are specified in the Supplementary data (Tables S1–S3). Food intake monitoring was conducted by using the TSE PhenoMaster system [7], demonstrating no significant changes due to the treatment of SEE and EMIQ (Figure S4).

For the intraperitoneal glucose tolerance test (GTT), D-glucose (2 g/kg body weight, Sigma, St. Louis, MO, USA) was injected intraperitoneally to each mouse, and glucose concentrations were measured as indicated. Nuclear magnetic resonance (NMR) scanning EchoMRI-700 (Echo Medical Systems) was used to measure the body composition. Ex vivo electron transport activity related to mitochondrial oxidative phosphorylation was evaluated by monitoring the reduction of triphenyltetrazolium chloride (TTC, Sigma, St. Louis, MO, USA), as described previously [19]. Hematoxylin and eosin (H&E) staining was performed using paraffin sections, as described previously [20]. Cell diameters in WAT were determined from the H&E stained paraffin section images.

### 2.4. Analysis of Mitochondrial Function

Oxygen concentrations and oxygen consumption rates (OCRs) were measured by the Seahorse XF Analyzers (Agilent, Santa Clara, CA, United States) [7,17]. OCRs were normalized according to protein concentrations. Uncoupled respiration was calculated by subtracting the rotenone and antimycin A-induced OCR from the oligomycin A-induced OCR. ATP-related respiration was calculated by subtracting the oligomycin A-induced OCR from the basal OCR.

### 2.5. Western Blot Analysis

Western blotting was performed, as previously described [20]. Western blot analysis was performed using primary antibodies against phosphorylated hormone-sensitive lipase (p-HSL Ser660, rabbit, Cell Signaling), hormone-sensitive lipase (HSL, rabbit, Cell Signaling), phosphorylated perilipin1 (p-PLIN1 Ser522, mouse, VALA science), perilipin1 (PLIN1, rabbit, Santacruz), beta-actin (mouse, Santacruz), phosphor-(Ser/Thr) protein kinase A substrates (p-PKA substrates, rabbit, Cell Signaling), sirtuin 1 (SIRT1, rabbit, Cell Signaling), Estrogen Receptor Alpha Antibody A300-498A (Estrogen receptor alpha (ERα), rabbit, Bethyl laboratories INC.), adipose triglyceride lipase (ATGL, rabbit, Cell Signaling), phosphor- cAMP-response element binding protein (p-CREB ser133, rabbit, Cell Signaling), cAMP-response element binding protein (CREB, rabbit, Cell Signaling), uncoupling protein 1 (UCP1, rabbit, Alpha Diagnostic International), total oxphos rodent WB antibody cocktail (mouse, Abcam: ATP synthase subunit alpha, mitochondrial (ATP5A), succinate dehydrogenase [ubiquinone] iron–sulfur subunit, mitochondrial (SDHB), NADH dehydrogenase [ubiquinone] 1 beta subcomplex subunit 8, mitochondrial (NDUFB8), cytochrome b-c1 complex subunit 2, mitochondrial (UQCRC2)), and α/β tubulin (rabbit, Cell Signaling). Blots were visualized with Super Signal West Dura Substrate (Pierce, Invitrogen).

### 2.6. Statistical Analysis

GraphPad Prism 5 software (GraphPad Software, La Jolla, CA, USA) was used for statistical analysis. Statistical significance between two groups was determined by unpaired t-test. Data are presented as mean ± standard errors of the means (SEMs). Comparisons among multiple groups were performed using a two-way analysis of variance (ANOVA), with Bonferroni post hoc tests to determine *p* values.

## 3. Results

### 3.1. SEE Reduced Triglyceride Content and Increased Lipolysis in Adipocytes In Vitro

**Figure 1:**
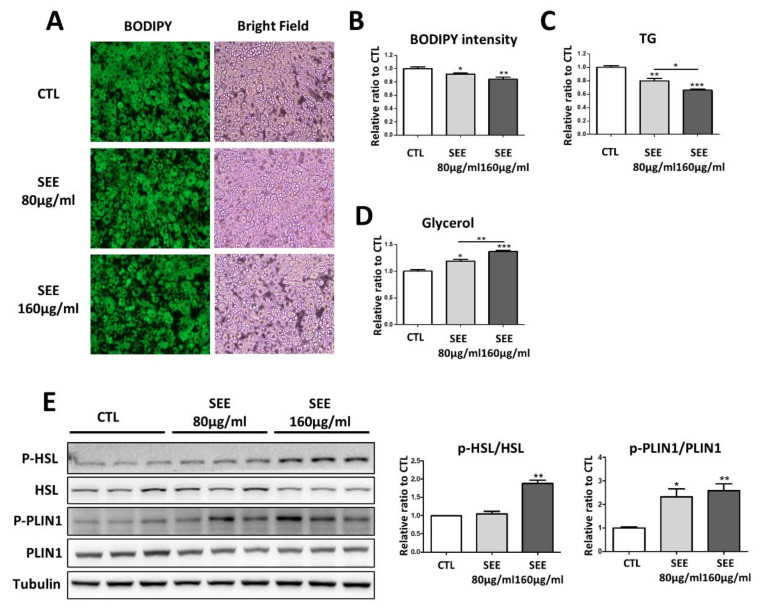
Effects of in-vitro soybean embryo extract treatment on lipid content in adipocytes differentiated from C3H10T1/2 cells. (**A**) Representative images of boron dipyrromethene fluorophore (BODIPY) staining of adipocytes differentiated from C3H10T1/2 cells, treated with soybean embryo extract (SEE) (80 µg/mL, 160 µg/mL) for 24 h, along with bright filed images; (**B**) quantification of BODIPY staining intensity of A. Groups of 160 µg/mL SEE treated have the lowest BODIPY intensity (*n* = 6, means ± SEM, * *p* < 0.05, ** *p* < 0.01, *** *p* < 0.001); (**C**) intracellular triglyceride (TG); (**D**) glycerol levels in conditioned media (*n* = 6, means ± SEM, * *p* < 0.05, ** *p* < 0.01, *** *p* < 0.001); (**E**) immunoblot analysis of phosphorylated hormone-sensitive lipase(p-HSL), hormone-sensitive lipase(HSL), phosphorylated perilipin1(p-PLIN1), perilipin1(PLIN1) and tubulin in adipocytes differentiated from C3H10T1/2 cells treated with vehicle (CTL), SEE (80 µg/mL) and SEE (160 µg/mL) for 24 h. (*n* = 6, means ± SEM, ** *p* < 0.01).

To investigate the effect of SEE on in vitro lipolysis, we treated fully differentiated C3H10T1/2 adipocytes with SEE for 24 h. Neutral lipid staining by BODIPY and intracellular TG assay indicated that SEE treatment significantly reduced lipid content in adipocytes (Figure 1A–C). As an indicator of TG hydrolysis, glycerol content in conditioned media was increased by SEE treatment (Figure 1D). Next, we examined the expression and phosphorylation levels of HSL and PLIN1 because HSL and PLIN1 are regulated by PKA-dependent phosphorylation. SEE treatment increased the phosphor-form of HSL and PLIN1 (Figure 1E), indicating that SEE increased PKA-dependent cytosolic lipolysis.

### 3.2. SEE Upregulated Mitochondrial Content and Respiration via PKA Signaling in Adipocytes In Vitro

**Figure 2:**
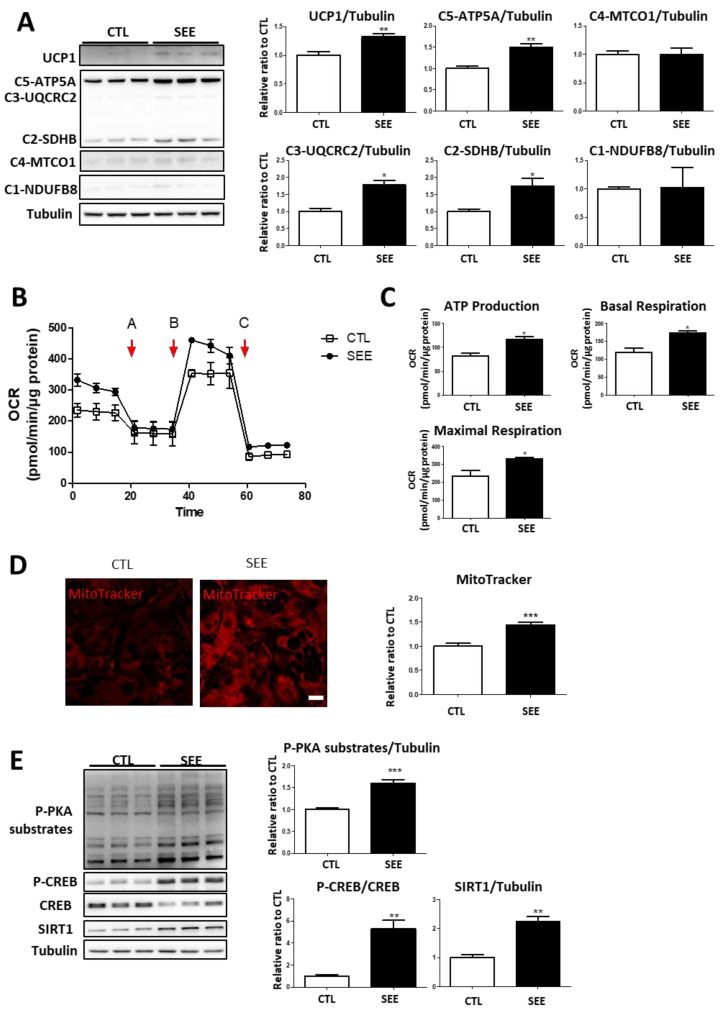
Effects of in vitro SEE treatment on mitochondrial function and PKA signaling in adipocytes. (**A**) Immunoblot analysis of uncoupling protein 1(UCP1) and mitochondrial proteins involved in oxidative phosphorylation in adipocytes differentiated from C3H10T1/2 cells treated with vehicle (CTL) and SEE (160 µg/mL) for 24 h; (**B**) analysis of oxygen consumption rate (OCR) of adipocytes differentiated from C3H10T1/2 cells treated with vehicle (CTL), and SEE (160 µg/mL) for 24 h with a series of treatments of indicated drugs (Oligomycin, Carbonyl cyanide-4 (trifluoromethoxy)phenylhydrazone (FCCP), rotenone and antimycin A). Arrows of (B) indicate the time of the treatments; (**C**) comparisons of basal mitochondrial respiration and ATP-linked respiration between cells treated with vehicle and SEE; (**D**) MitoTracker staining of adipocytes differentiated from C3H10T1/2 cells treated with vehicle (CTL) and SEE (160 µ g/mL) for 24 h. size bar = 20 µm (*n* = 6, means ± SEM, * *p* < 0.05, ** *p* < 0.01, *** *p* < 0.001); (**E**) immunoblot analysis of p-CREB, CREB, SIRT1, and p-PKA substrates in adipocytes differentiated from C3H10T1/2 cells treated with vehicle (CTL) and SEE (160 µg/mL) for 24 h.

As we observed an increase in phosphorylation levels of HSL by SEE, we hypothesized that SEE might induce a brown adipocyte phenotype and increase mitochondrial activity via the PKA signaling-dependent pathways. Expression levels of UCP1 and mitochondrial proteins involved in oxidative phosphorylation (ATP5A, UQCRC2, and SDHB) were significantly increased by SEE treatment (Figure 2A). Moreover, SEE treatment increased phosphorylation levels of CREB. In addition, we used antibody against phospho-PKA substrates to detect peptides and proteins containing a PKA substrate motif (phospho-serine/threonine residue with arginine at the –3 position), and demonstrated that treatment with SEE increased the phosphorylation levels of PKA downstream targets (Figure 2E). SIRT1, which is known to regulate mitochondrial biogenesis [21], was upregulated by SEE treatment (Figure 2E).

To determine whether SEE treatment induces an increase in mitochondrial activity, we examined the oxygen consumption rate (OCR) using C3H10T1/2 adipocytes. SEE treatment increased basal and maximal OCR, and OCR was related to ATP production (Figure 2B,C). Moreover, SEE treatment increased mitochondrial membrane potential, as determined by MitoTracker staining (Figure 2D).

### 3.3. SEE and EMIQ Had Synergistic Effects on Mitochondrial Content and Activity in Adipose Tissue In Vivo

**Figure 3:**
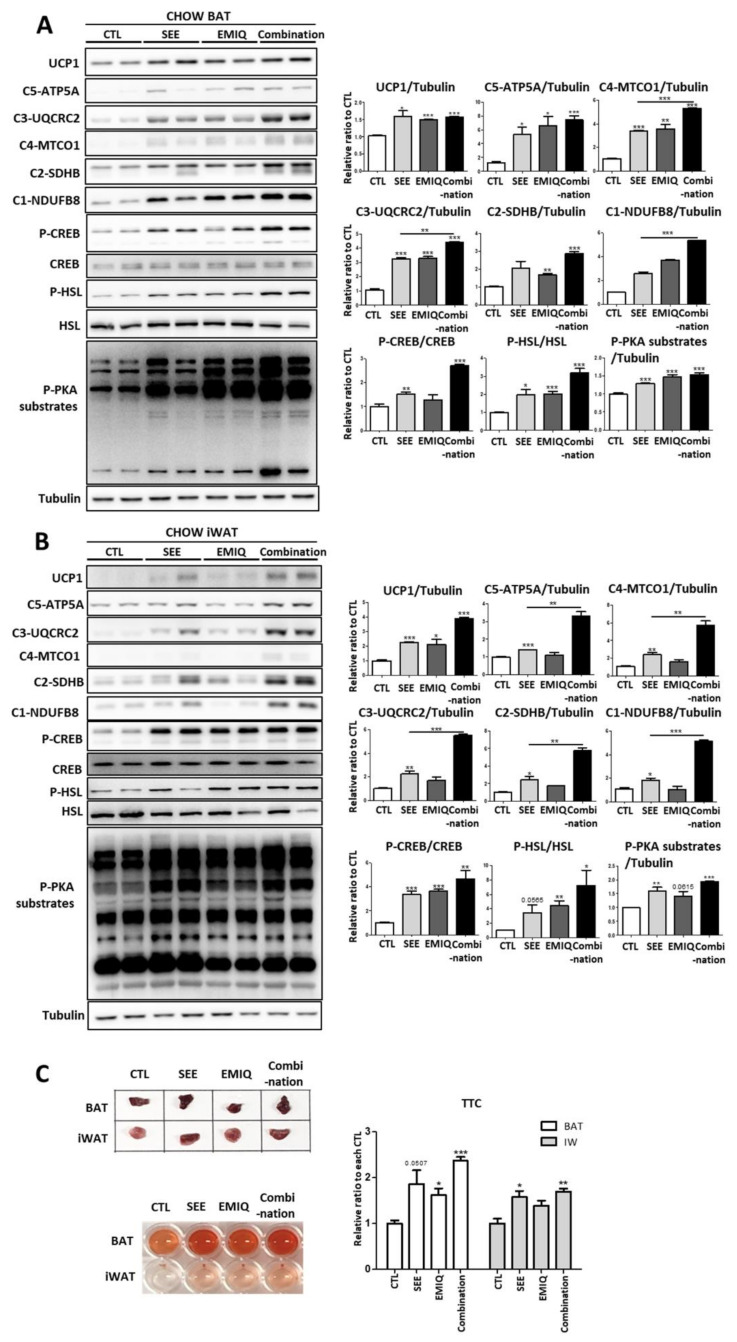
Effects of in vivo SEE, enzymatically modified isoquercitrin (EMIQ), and co-treatment (SEE + EMIQ) treatment on mitochondrial content and PKA signaling in adipose tissues. (**A**,**B**) Immunoblot analysis of UCP1 and proteins involved in mitochondrial oxidative phosphorylation, and PKA signaling in brown adipose tissue (BAT) and inguinal white adipose tissue (iWAT) of mice treated with vehicle (CTL), SEE (400 mg/kg), EMIQ (100 mg/kg) and co-treatment (Combination: SEE 400 mg/kg + EMIQ 100 mg/kg) for two weeks. (*n* = 6, means ± SEM, * *p* < 0.05, ** *p* < 0.01, *** *p* < 0.001) (**C**) Mitochondrial respiration in BAT and iWAT of male mice treated with distilled water as vehicle, SEE, EMIQ, and combination (SEE + EMIQ) for two weeks (mean  ±  SEM; *n* = 6, * *p* < 0.05, ** *p* < 0.01, *** *p* < 0.001) as determined by reduction of the electron acceptor dye triphenyltetrazolium chloride (TTC).

Next, we examined the in vivo effects of SEE on the mitochondrial contents and activity of adipose tissue. In this study, we used EMIQ as a positive control that possesses a WAT browning effect [16]. We also examined the synergistic effects of co-treatment with EMIQ and SEE. Each group of mice was treated with SEE (400 mg/kg), EMIQ (100 mg/kg), or a combination of two reagents (SEE 400 mg/kg, EMIQ 100 mg/kg) for two weeks. In BAT and WAT, single and combination treatment with SEE and EMIQ significantly increased UCP1 expression and mitochondrial proteins related to oxidative phosphorylation (Figure 3A,B). The combination treatment with SEE and EMIQ showed an additive effect on the increase in several mitochondrial proteins (MTCO1, UQCRC2, NDUFB8 in BAT: Figure 3A; ATP5A, MTCO1, UQCRC2, SDHB, NDUFB8 in iWAT: Figure 3B). Consistent with data from the in vitro studies, treatment of SEE, EMIQ, and the combination of two reagents increased phosphorylation levels of PKA downstream proteins (PKA substrates, CREB, and HSL) (Figure 3A,B). Next, we examined the oxidative capacity of adipose tissue obtained from SEE- and EMIQ-treated mice. This functional analysis by TTC staining demonstrated that mitochondrial electron transport was upregulated by SEE (400 mg/kg) and EMIQ (100 mg/kg) in BAT and iWAT (Figure 3C).

### 3.4. SEE and EMIQ Had an Anti-Obesity Effect in High-Fat Diet-Induced Obesity Mouse Models

**Figure 4:**
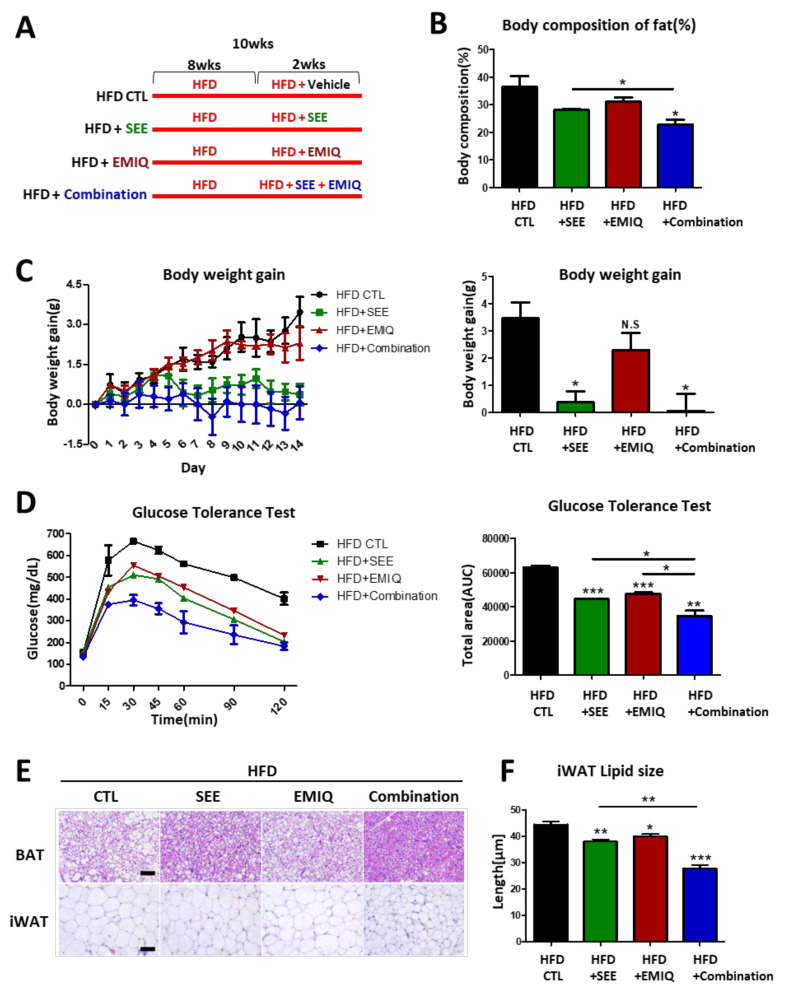
Effects of in vivo SEE, EMIQ, and co-treatment (SEE + EMIQ) treatment on body weight and glucose tolerance of mice fed with a high fat diet. (**A**) Study design for 8 weeks of high fat diet feeding and two weeks of high-fat diet(HFD) + Control vehicle, SEE (400 mg/kg), EMIQ (100 mg/kg) and Combination (SEE 400 mg/kg + EMIQ 100 mg/kg) treatment. (**B**) Body composition of fat percentage. (**C**) Body weight gain monitoring while two weeks of HFD + SEE, EMIQ, Combination (SEE + EMIQ) treatment. (**D**) Glucose tolerance test. (**E**) Hematoxylin and eosin(H/E) staining of paraffin section of BAT and iWAT obtained from the HFD-fed mice treated with Control vehicle, SEE, EMIQ, Combination (SEE+EMIQ) for two weeks (size bars = 50 μm). (**F**) Adipocyte size analysis of H/E stained paraffin sections of inguinal WAT (*n* = 6, means ± SEM, * *p* < 0.05, ** *p* < 0.01, *** *p* < 0.001).

To assess the anti-obesity effects of SEE and EMIQ, we used a high-fat diet(HFD)-induced obesity mouse model. Mice were fed a HFD for 8 weeks and each group was treated with SEE, EMIQ, or a combination of SEE with EMIQ for two weeks, with the continuation of HFD feeding (Figure 4A). After two weeks of treatment, SEE single treatment and combination treatment with EMIQ significantly reduced body fat composition (%) and body weight gain (Figure 4B,C). Similarly, adipocyte diameters in WAT were reduced by SEE single treatment and combination treatment with EMIQ (Figure 4E,F). Glucose tolerance was improved by single and combination treatment (Figure 4D). Combination treatment with SEE and EMIQ showed an additive effect on improved glucose tolerance (Figure 4D).

**Figure 5:**
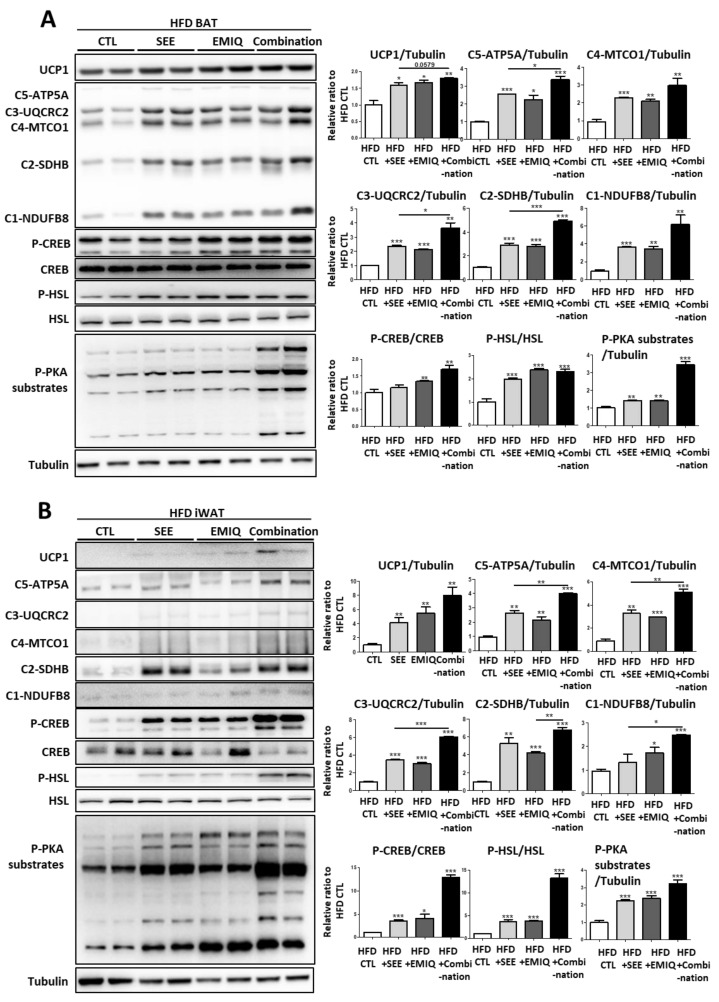
Effects of in vivo SEE, EMIQ, and combination (SEE + EMIQ) treatment on mitochondrial content and browning marker expression in adipose tissues of high fat diet-induced obesity model. (**A**,**B**) Immunoblot analysis of UCP1, and proteins involved in mitochondrial oxidative phosphorylation, and PKA signaling (**A**) in BAT and (**B**) iWAT of mice treated with vehicle (CTL), SEE (400 mg/kg), EMIQ (100 mg/kg), and combination (SEE 400 mg/kg + EMIQ 100 mg/kg) for two weeks (*n* = 6, means ± SEM, * *p* < 0.05, ** *p* < 0.01, *** *p* < 0.001).

Consistent with data from mice fed normal chow diets, treatment with SEE and EMIQ significantly increased mitochondrial content, and phosphorylation levels of PKA downstream proteins and co-administration showed synergic upregulation in several mitochondrial proteins in BAT and WAT (Figure 5A,B).

**Figure 6:**
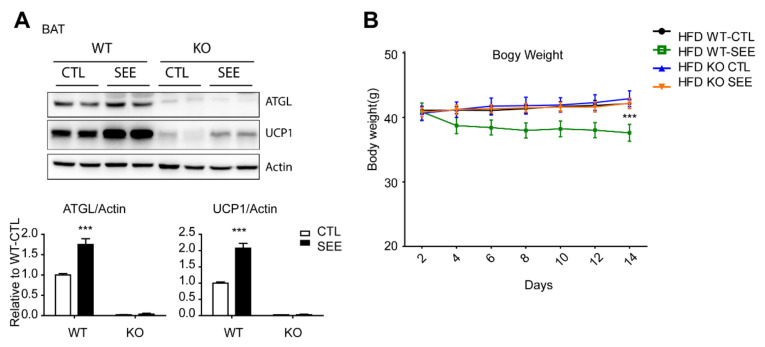
Adipocyte-specific ATGL knockout attenuates effects of SEE on body weight in high fat diet induced obesity model. (**A**) Immunoblot analysis of ATGL and UCP1 in BAT of WT controls and adipocyte-specific adipose triglyceride lipase (ATGL) knockout (KO) mice, fed HFD for 8 weeks and treated with vehicle (CTL) and SEE (400 mg/kg) for two weeks. (**B**) Body weight monitoring of WT controls and adipocyte-specific ATGL KO mice during SEE treatment (*n* = 6, means ± SEM, *** *p* < 0.001).

Finally, to determine whether the anti-obesity effect of SEE required activation of cytosolic lipolysis, we used adipocyte-specific adipose triglyceride lipase (ATGL) knockout (KO) mouse models. We used the ATGL KO model as ATGL is the cytosolic lipase that initiates the hydrolysis of TG and its activity is regulated by abhydrolase domain containing 5 (ABHD5) dissociated from PLIN1 by PKA-dependent phosphorylation [13] (Figure S6B). ATGL knockout in adipose tissue was confirmed by western blotting (Figure 6A). Although phosphorylation levels of CREB an HSL were not affected by abrogation of ATGL (Figure S6A), ATGL KO reduced UCP1 expression in BAT, consistent with previous studies [22]. Treatment with SEE did not affect the expression levels of UCP1or reduced body weight (Figure 6A,B). Collectively, these data indicate that activation of cytosolic lipolysis is the major mechanism of the anti-obesity effect of SEE.

## 4. Discussion

BAT is a thermoregulatory tissue known to contribute to beneficial changes that improve metabolic profiles and energy expenditure [4]. Thus, it has gained significant attention for its therapeutic potential in the treatment of obesity-related metabolic diseases [4]. Increasing BAT activity and inducing brown adipocyte phenotype in WAT (i.e., WAT browning) can be achieved by thermogenic stimuli, and thus, efforts have been made to develop molecules that mimic thermogenic stimuli for candidate anti-obesity/diabetes therapeutics. In this regard, our study demonstrated that SEE reduced body weight and improved insulin sensitivity in HFD-fed mice, in part by upregulation of brown adipocyte metabolism, and an increase in WAT browning. Mechanistically, SEE upregulated mitochondrial oxidative respiration and thermogenic/lipolytic gene expression through the cAMP/PKA-dependent signaling pathway in BAT and WAT. In addition, SEE effects required cytosolic lipase ATGL for this anti-obesity effect, as shown by adipocyte-specific ATGL KO mouse study. Although the current study focused on the ATGL-mediated mechanisms as one of PKA-downstream pathways, we do not exclude the possibility that other PKA-downstream targets contribute to the anti-obesity effects of SEE. Further studies using genetic KO models and pharmacological inhibitors of PKA signaling will be required to determine the molecular mechanisms of SEE-mediated modulation of lipid metabolism.

Recently, naturally occurring compounds with browning effects have been intensively investigated. An accumulating body of evidence has suggested that phytochemicals that activate BAT activity include flavonoids, terpenoids, and alkaloids, while multiple signaling pathways are involved in these effects [1,23]. SEE has been used as the raw material for functional health food containing isoflavones, mainly daidzein, glycitein, and genistein [8]. Consumption of SEE has the positive effects of reducing excess body weight, improving hyperinsulinemia, and the risk factors related to obesity [9]. In addition, the anti-obesity effects of individual compounds in SEE have been investigated in relation to lipid metabolism [24]. For example, genistin is known to inhibit lipid accumulation in 3T3-L1 adipocytes [10], and the mix of daidzin and glycitin treatment in high-fat diet C57BL/6J mice lower body weight [24]. Chronic treatment with daidzein reduced weight gain and fat content in the liver [25]. However, the molecular mechanisms of each compound’s underlying anti-obesity effects are not clear. Further studies will be required to elucidate the effect of each ingredient on lipid metabolism and their molecular mechanisms.

Isoflavones are nonsteroidal compounds with phytoestrogen activity resulting from the structural resemblance of natural estrogen [26]. Previous studies suggest the roles of phytoestrogens in alleviating menopausal symptoms [27]. As menopausal symptoms are associated with abdominal obesity, it would be informative to investigate the effects of SEE on menopause-associated metabolic dysfunction in female in vivo models. Although the expression levels of estrogen receptor alpha were not affected by SEE treatment (Figure S7), further study is required to understand the effects of SEE on estrogen receptor-mediated downstream signaling pathways in adipocytes.

We investigated the co-treatment effect with SEE and EMIQ, and demonstrated its synergistic effects on the improvement of metabolic function. Previous reports indicated the browning effects of EMIQ through AMPK activation [16]. We speculated that co-treatment may target multiple pathways that regulate lipid metabolism to improve the metabolic profile synergistically. In addition to its effect on adipose tissue, EMIQ has beneficial effects through the regulation of liver lipid metabolism and has therapeutic potential for the treatment of diabetes. For instance, isoquercitrin exerts anti-diabetic effects through the activation of hepatic glucose-6-phosphatase, which controls hyperglycemia in rodent models [28,29]. In this regard, to potentiate the beneficial effects of phytochemicals by targeting multiple pathways in major metabolic organs, combination therapy could be recommended for clinical use in the treatment of metabolic diseases.

## 5. Conclusions

In conclusion, this study demonstrates that SEE exerts anti-obesity effects by promoting BAT activity and WAT browning and exhibits a synergistic effect of co-treatment with EMIQ. Phytoestrogen could be developed to target adipose tissue metabolism to increase energy expenditure and ultimately treat obesity-related metabolic diseases.

## Supplementary Materials

The following are available online at https://www.mdpi.com/2218-273X/10/10/1394/s1, Table S1: Composition of the standard food and the obesity induction food, Table S2: CHOW diet, Table S3: High fat diet, Figure S1: Membrane images of western blot analysis used for Figure 1., Figure S2: Membrane images of western blot analysis used for Figure 2, Figure S3: Membrane images of western blot analysis used for Figure 3, Figure S4: Effects of SEE and EMIQ on food consumption, Figure S5: Membrane images of western blot analysis used for Figure 5, Figure S6: Immunoblot analysis of PKA signaling related markers in BAT of WT controls and adipocyte-specific ATGL KO mice, Figure S7: Immunoblot analysis of Estrogen Receptor Alpha in adipocytes differentiated from C3H10T1/2 cells.
