# Supplementary material for: Anti-Obesity Effects of Soybean Embryo Extract and Enzymatically-Modified Isoquercitrin
Source: Biomolecules. 2020 Sep 30;10(10):1394. doi: 10.3390/biom10101394 (PMC7601939; doi:10.3390/biom10101394)
Supplement: Supplementary file 1 [file biomolecules-10-01394-s001.pdf]

Supplementary Materials

Supplemental Tables

Table S1. Composition of the standard food and the obesity induction food

Calories(kcal%)	CHOW	HFD
Protein(%)	24.52	20
Fat(%)	12.41	60
Carbohydrate(%)	63.07	20

CHOW: Purina Lab. Rodent Chow (38057)

HFD: Research Diets. DIO Series Diets (D12492)

Table S2. CHOW diet (CHOW; Purina Laboratory Rodent Diet 38057)

Components*	Calories(%): Fat 12.41%, Protein 24.52%, Carbohydrate 63.07%	
Nutrients	Protein 20%	Arginine 1.26%, Cystine 0.37%, Glycine 0.87%, Histidine 0.5%, Isoleucine 0.82%, Leucine 1.47%, Lysine 1.01%, Methionine 0.33%, Phenylalanine 0.98%, Tyrosine 0.63%, Threonine 0.72%, Tryptophan 0.25%, Valine 0.91%
	Fat 4.5%	Linoleic Acid 1.10%, Linolenic Acid 0.12%, Arachidonic Acid 0.02%, Omega-3 Fatty Acids 1.11%
	Fiber 3.7%	
Minerals		Ash 7.25%, Calcium 1.2%, Phosphorus 0.62%, Phosphorus (non-phytate) 0.4%, Potassium 0.82%, Magnesium 0.16%, Sulfur 0.22%, Sodium 0.34%, Chlorine 0.47%, Fluorine 21.38 ppm, Iron 112.93 ppm, Zinc 128.85 ppm, Manganese 95.49 ppm, Copper 22.74 ppm, Cobalt 0.76 ppm, Iodine 1.42 ppm, Chromium 0 ppm, Selenium 0.32 ppm
Vitamins		Vitamin K 6.69 ppm, Thiamin Hydrochloride 11.02 ppm, Riboflavin 11.57 ppm, Niacin 217.7 ppm, Pantothenic Acid 88.72 ppm, Choline Chloride 3447.96 ppm, Folic Acid 13.6 ppm, Pyridoxine 11ppm, Biotin 0.15 ppm, B12 41 ppm, Vitamin A 28.03 IU/kg, Vitamin D3 (added) 4 IU/kg, Vitamin E 100 IU/kg

Table S3. High fat diet (HFD; Research Diets D12492) (Research Diets Inc., USA)

Components*	Calories(%): Fat 60%, Protein 20%, Carbohydrate 20%				
	Fat	Protein	Carbohydrate	Total	kcal/gm
gm%	34.9	26.2	26.3		5.24
kcal%	60	20	20	100	

Ingredient	gm	kcal
Casein, 80 Mesh	200	800
L-Cystine	3	12
Maltodextrin 10	125	500
Sucrose	68.8	275.2
Cellulose, BW200	50	0
Soybean Oil	25	225
Lard	245	2205
Mineral Mix S10026	10	0
DiCalcium Phosphate	13	0
Calcium Carbonate	5.5	0
Potassium Citrate, 1 H_2O	16.5	0
Vitamin Mix V10001	10	40
Choline Bitartrate	2	0
FD&C Blue Dye #1	0.05	0
Total	773.85	4057

Supplemental Figures

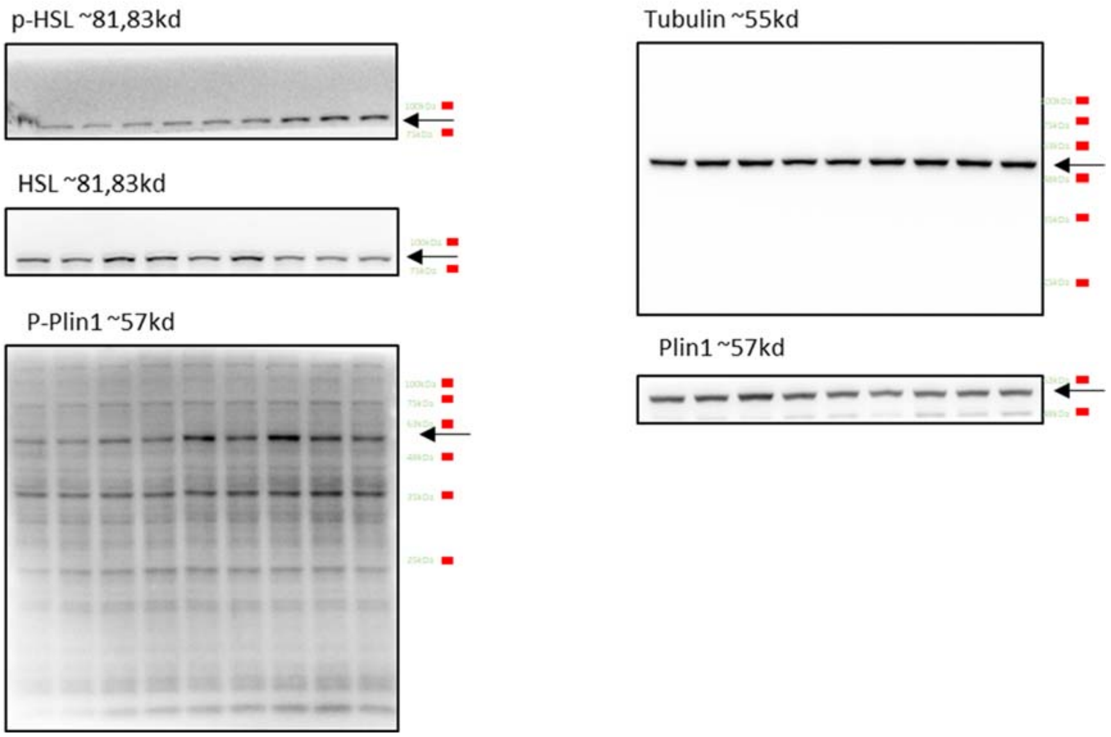

Figure S1. Membrane images of western blot analysis used for Figure 1.

Figure 2.A

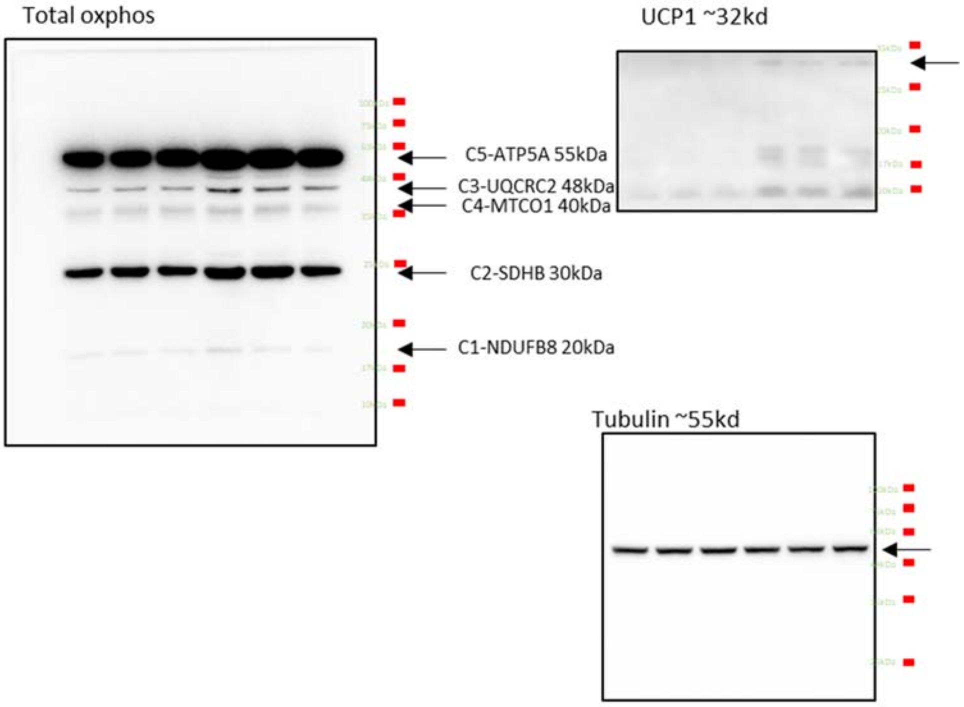

Figure 2.E

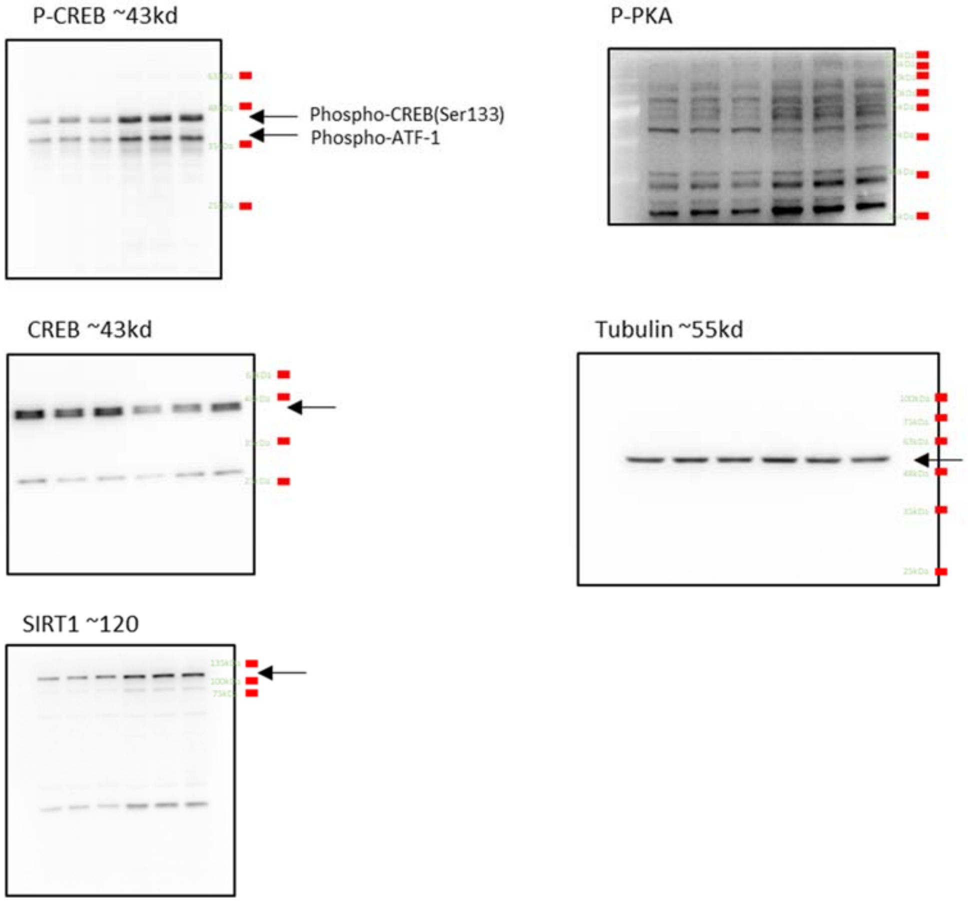

Figure S2. Membrane images of western blot analysis used for Figure 2.

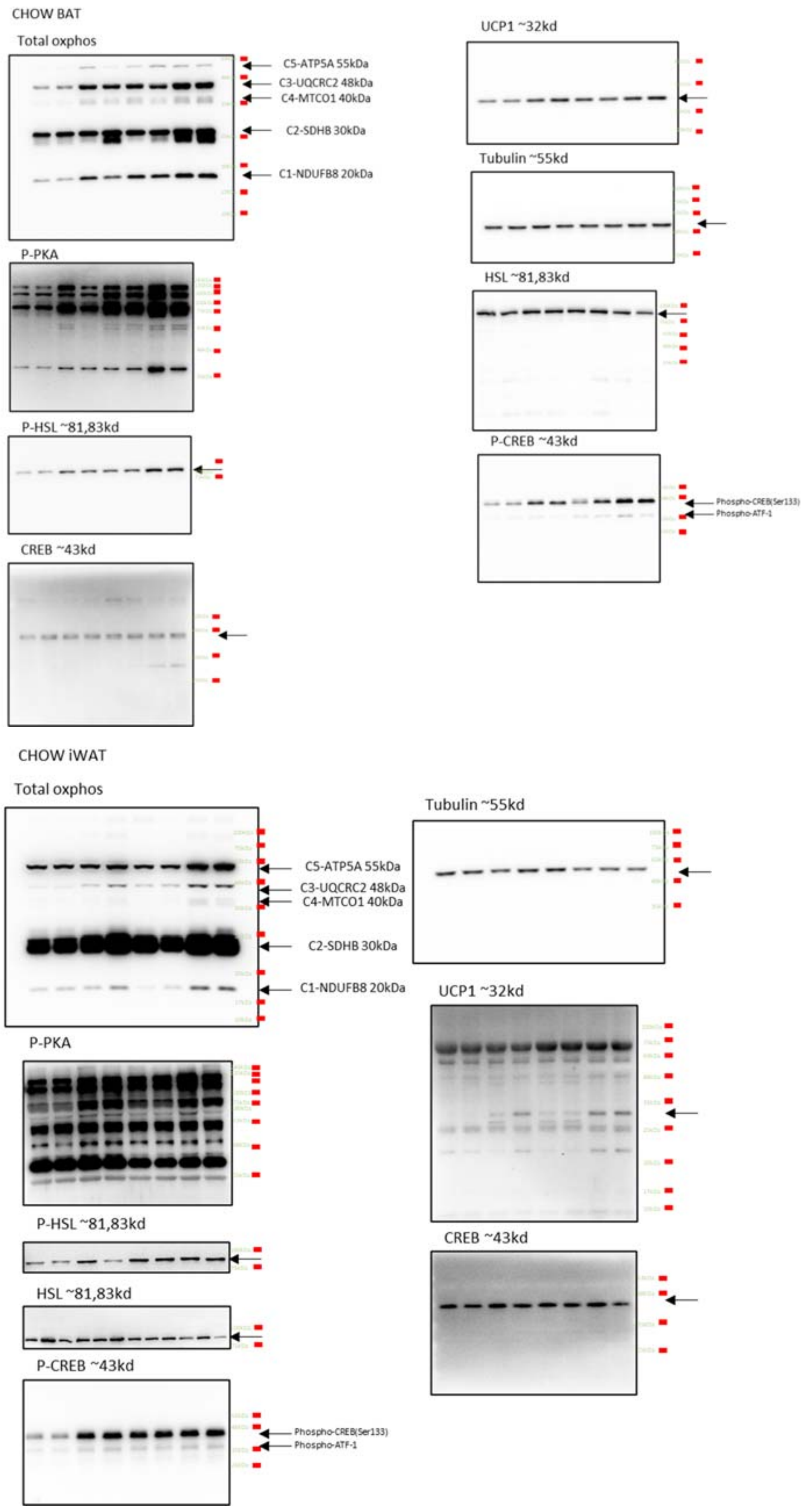

Figure S3. Membrane images of western blot analysis used for Figure 3.

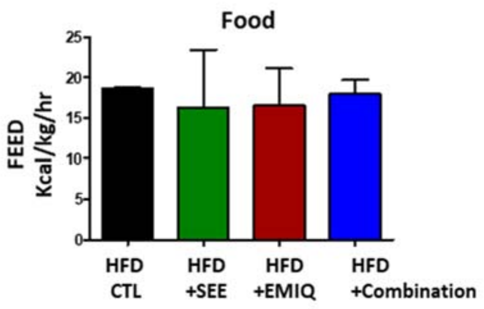

Figure S4. Effects of SEE and EMIQ on food consumption

Mice fed with 8 weeks of HFD were treated with SEE (400mg/kg/day), EMIQ (100mg/kg/day), or SEE/EMIQ for 2 weeks. Food consumption was monitored for 48 hours after the last dose by using TSE PhenoMaster system.

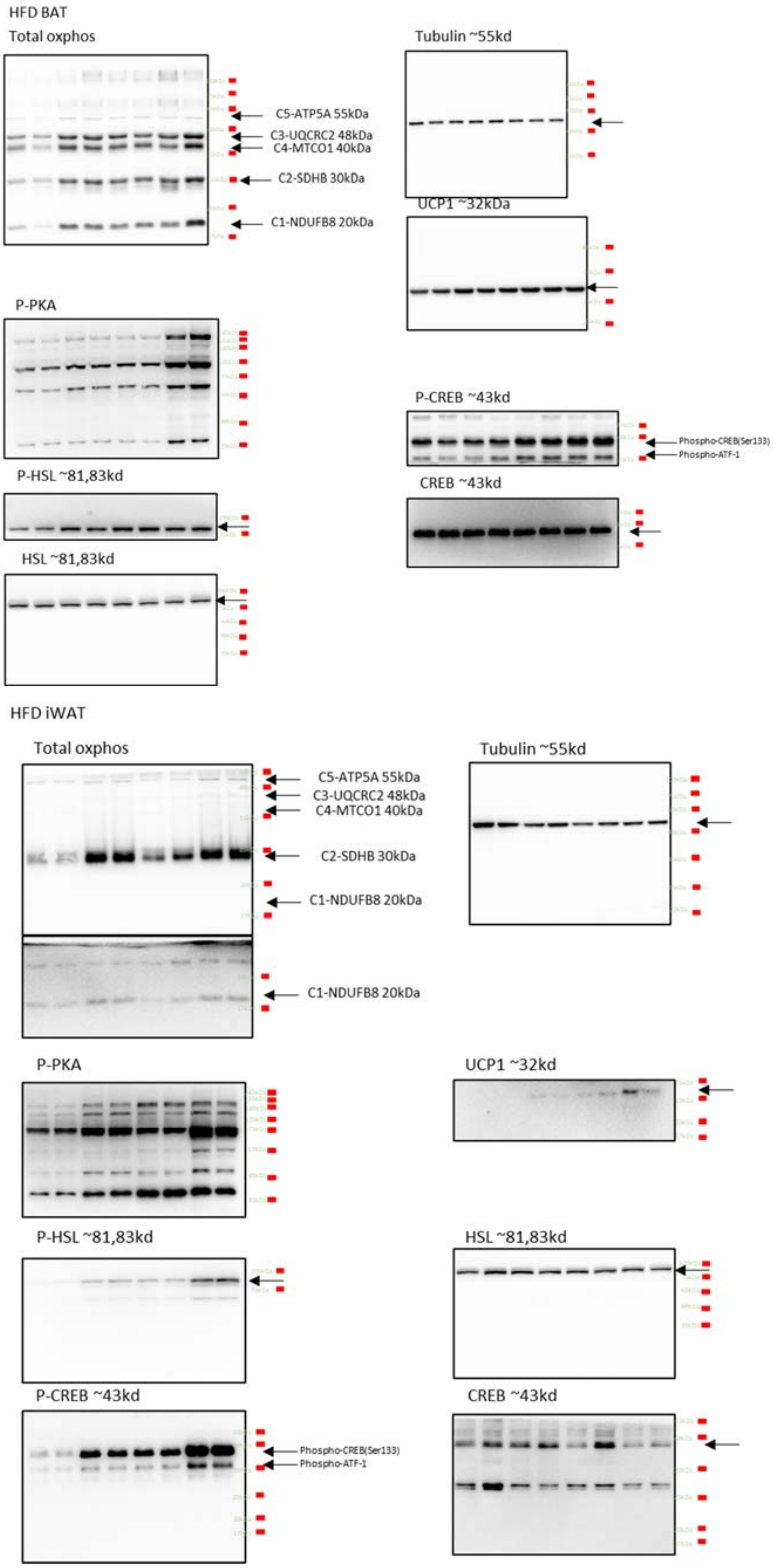

Figure S5. Membrane images of western blot analysis used for Figure 5.

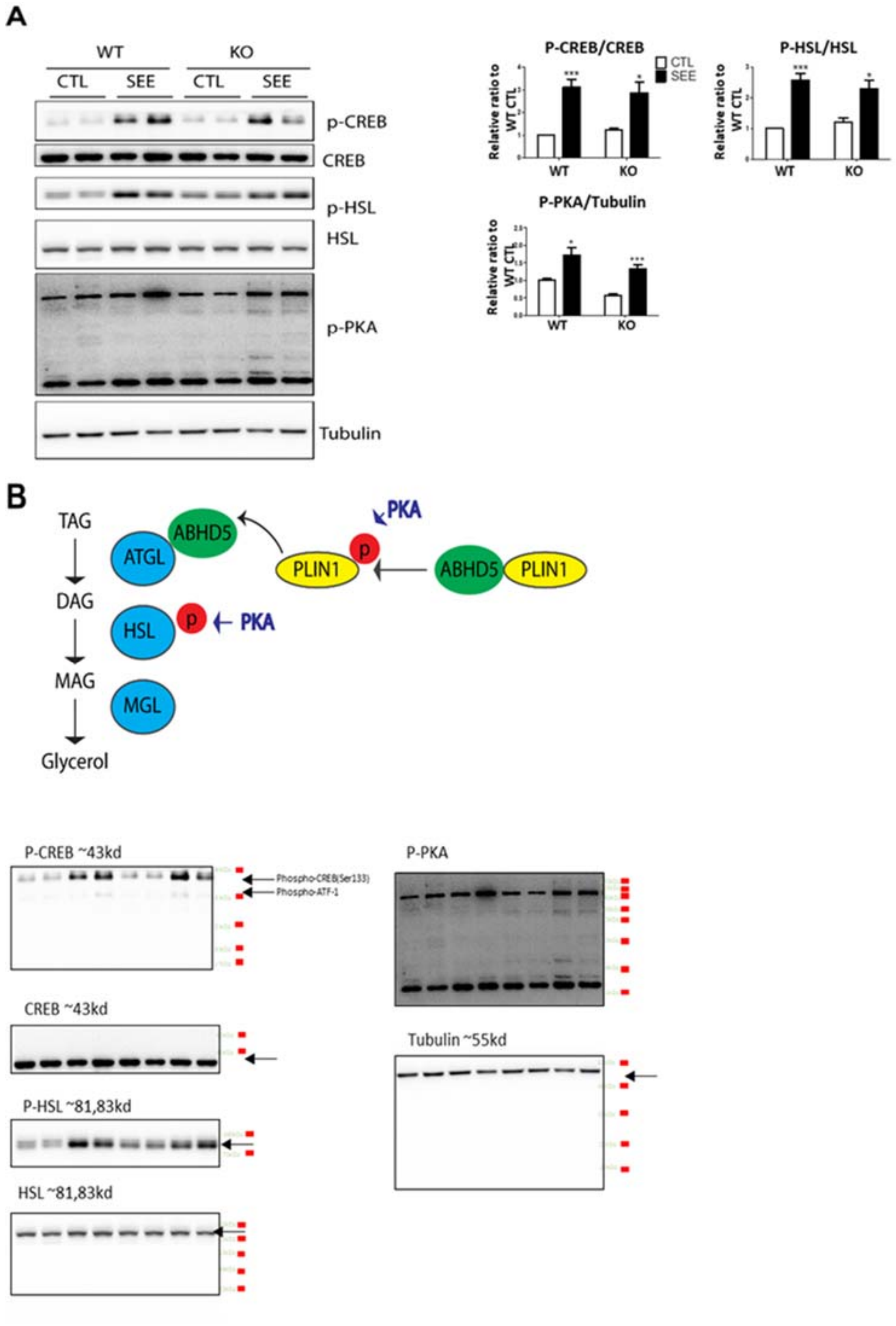

Figure S6. Effects of SEE on phosphorylation levels of CREB and HSL in BAT of adipocyte-specific ATGL knockout mice.

(A) Immunoblot analysis of proteins involved in PKA signaling (CREB, HSL and PKA substrates) in BAT of WT controls and adipocyte-specific ATGL KO mice, fed HFD for 8 weeks and treated with vehicle (CTL) and SEE (400mg/kg) for 2 weeks. (n = 6, means ± SEM, * $p < 0.05$, ** $p < 0.01$, *** $p < 0.001$) with membrane images of western blot analysis used for Figure S6. (n=6, means ± SEM, * $p < 0.05$, ** $p < 0.01$, *** $p < 0.001$).

(B) Diagram of PKA signaling.

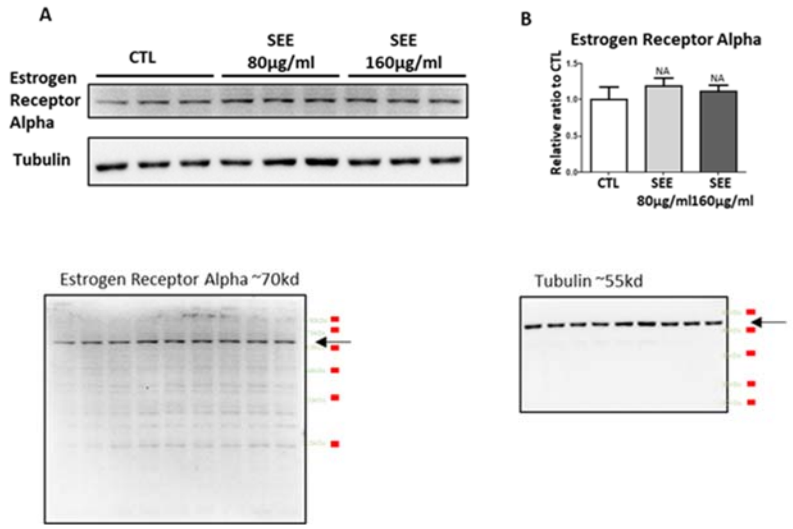

Figure S7. (A,B) Immunoblot analysis of Estrogen Receptor Alpha in adipocytes differentiated from C3H10T1/2 cells treated with Control vehicle (CTL), SEE (80μg/ml) and SEE (160μg/ml) for 24 h. (n=3, means ± SEM, * $p < 0.05$, ** $p < 0.01$, *** $p < 0.001$) and Membrane images of western blot analysis used for Figure S7.
